# Supplementary figures and images for: Multi-axial strain mapping to characterise structure and material properties of the human hip capsule
Source: PLoS One. 2026 Mar 10;21(3):e0343718. doi: 10.1371/journal.pone.0343718 (PMC12974790; doi:10.1371/journal.pone.0343718)

1 S1 Appendix. Opto-mechanical characterisation device parts lists and assembly

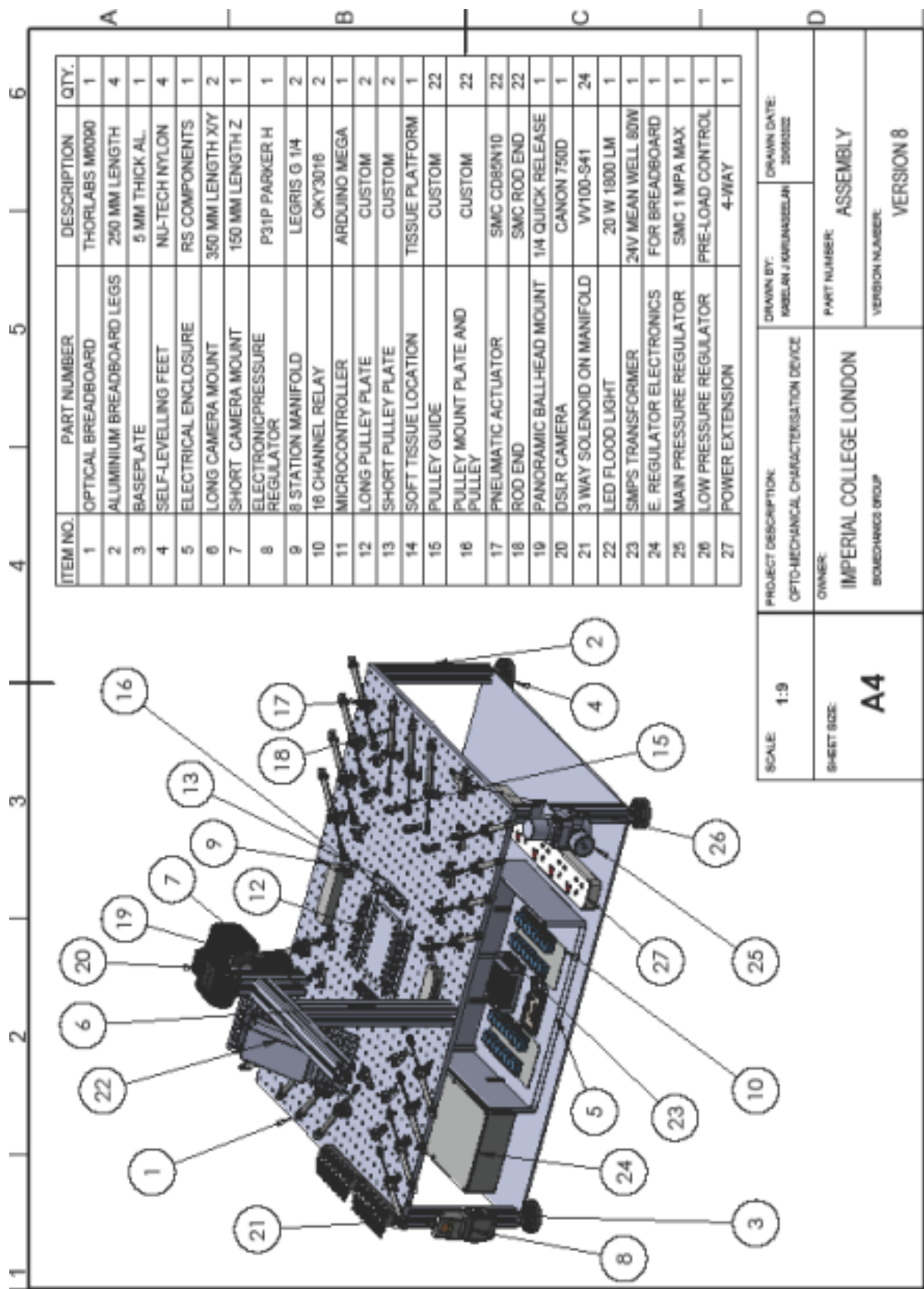

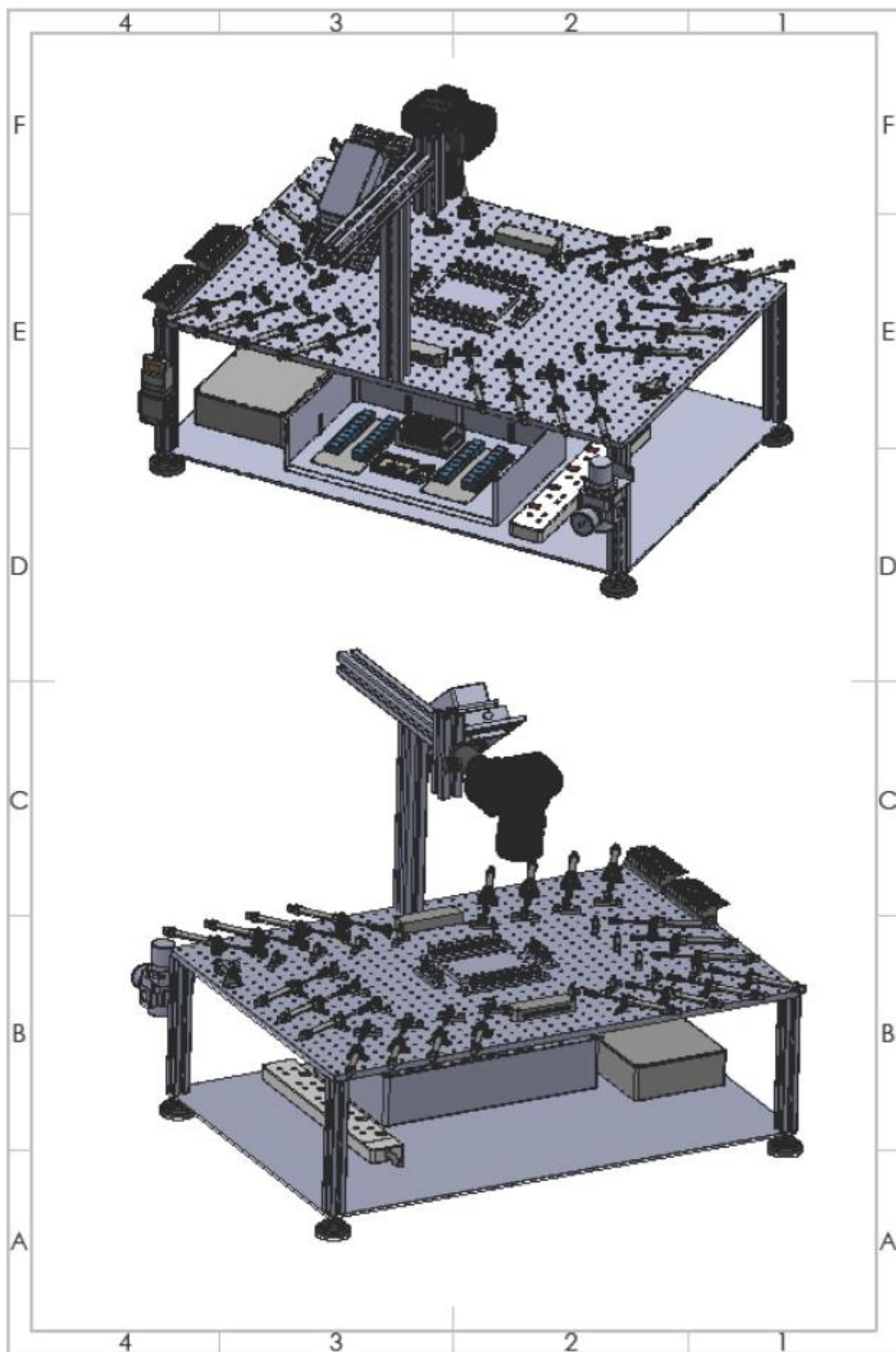

Supplement: S1 Appendix — (PDF) [file pone.0343718.s001.pdf]

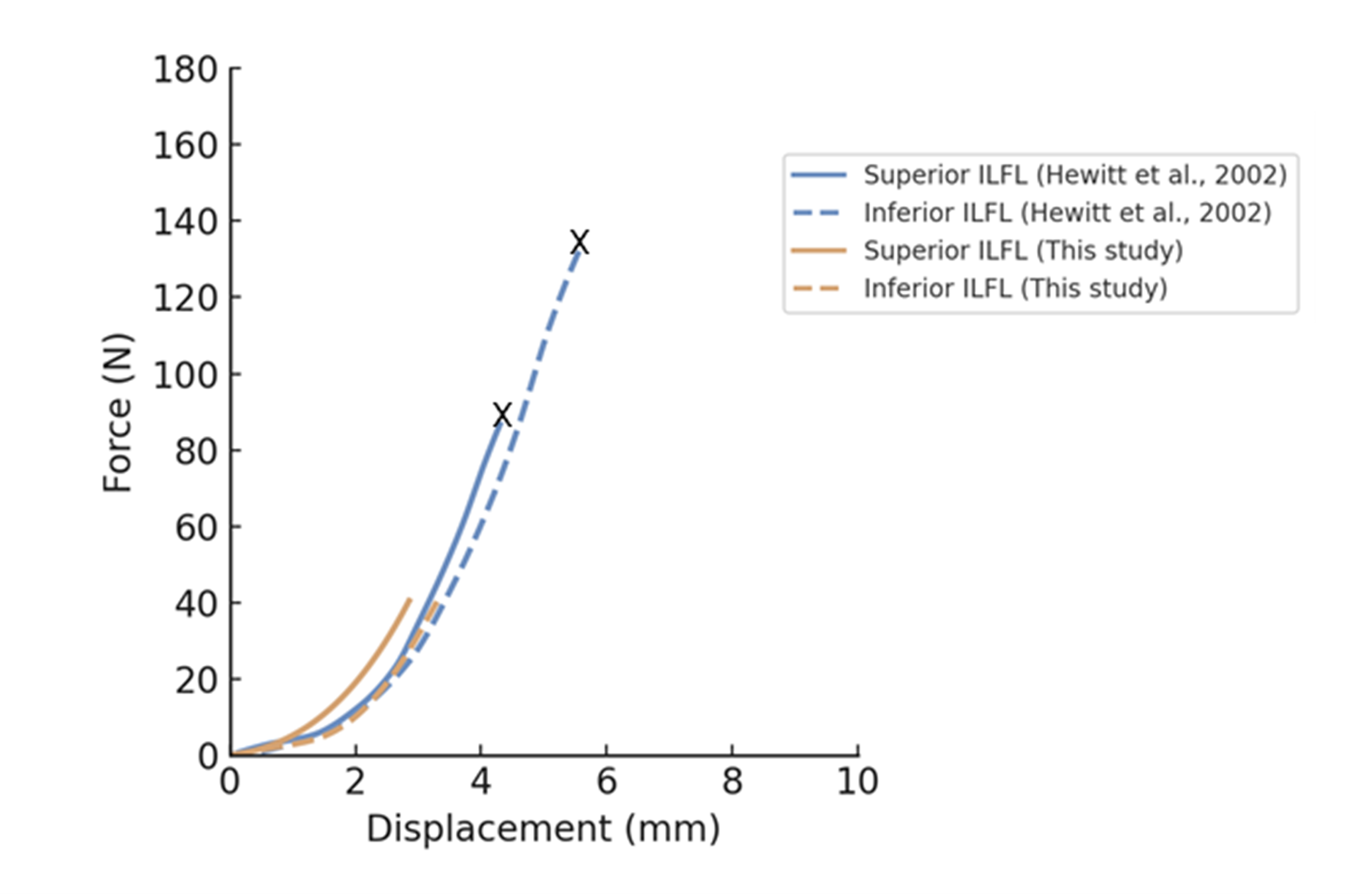

Supplement: S1 Fig — The ‘X’ markers indicate the approximate failure region from Hewitt et al. All loads applied in this study lie within the initial linear region and remain well below reported failure levels. (TIF) [file pone.0343718.s004.tif]

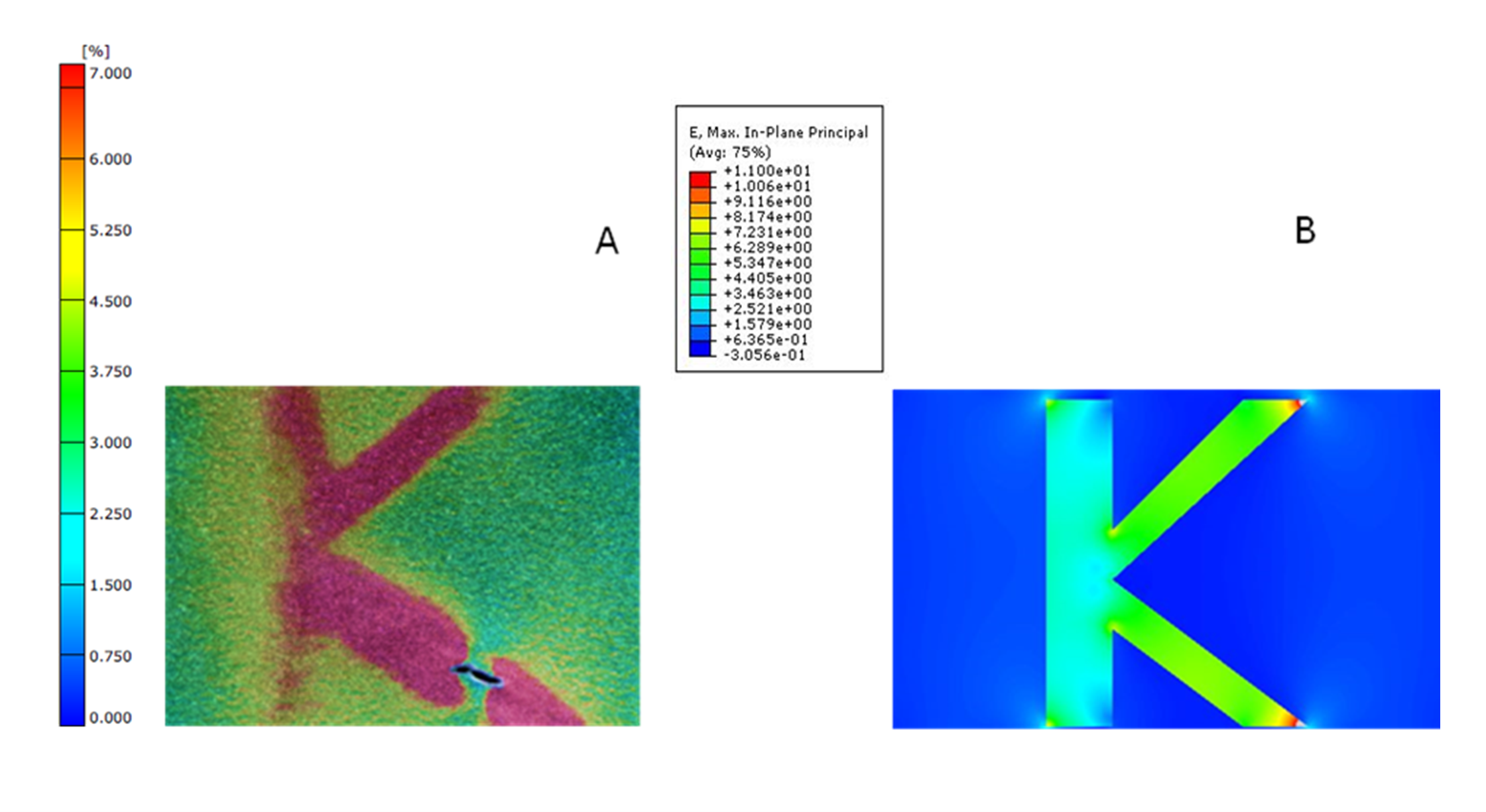

Supplement: S2 Fig — (TIF) [file pone.0343718.s005.tif]

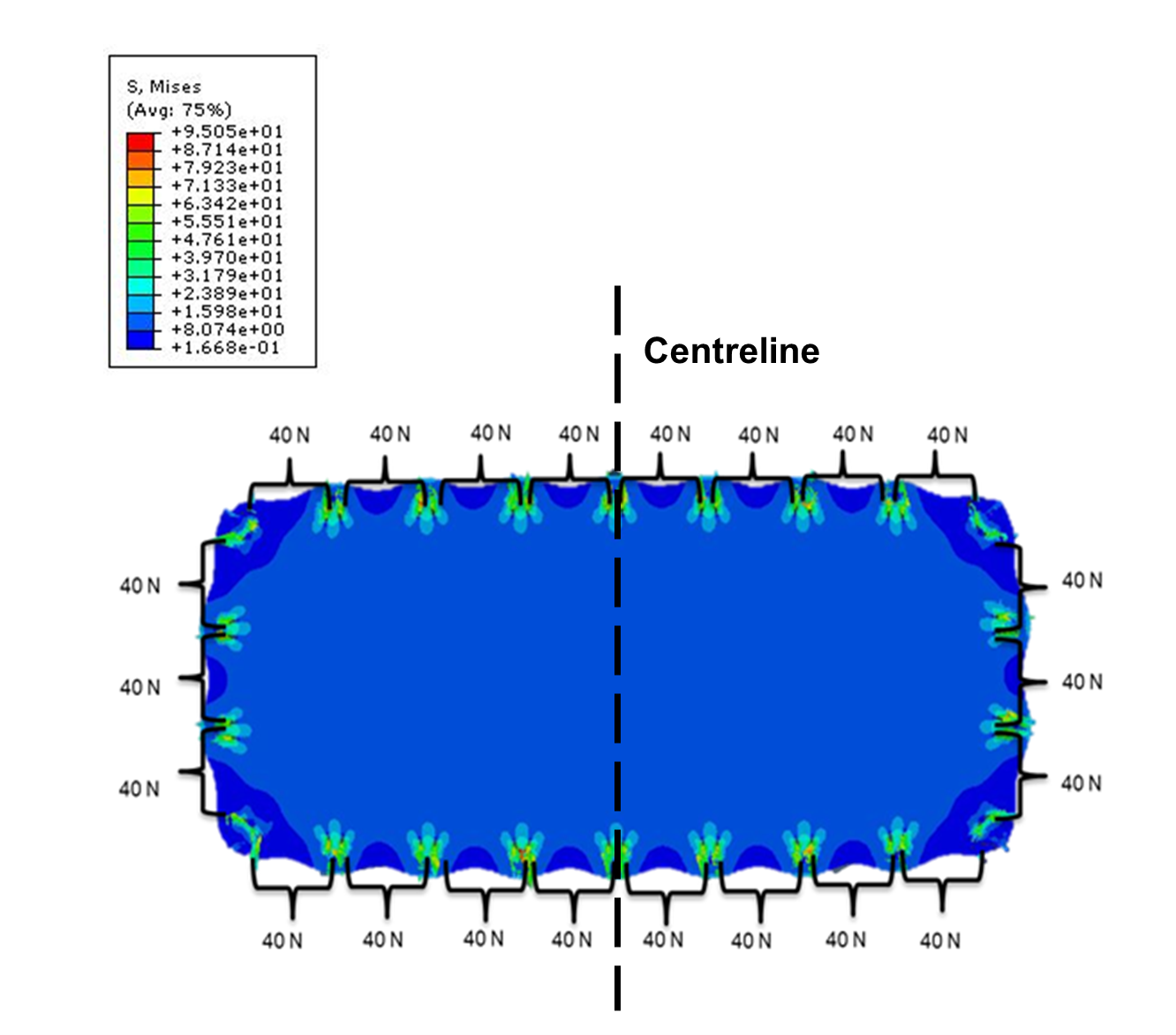

Supplement: S3 Fig — (TIF) [file pone.0343718.s006.tif]

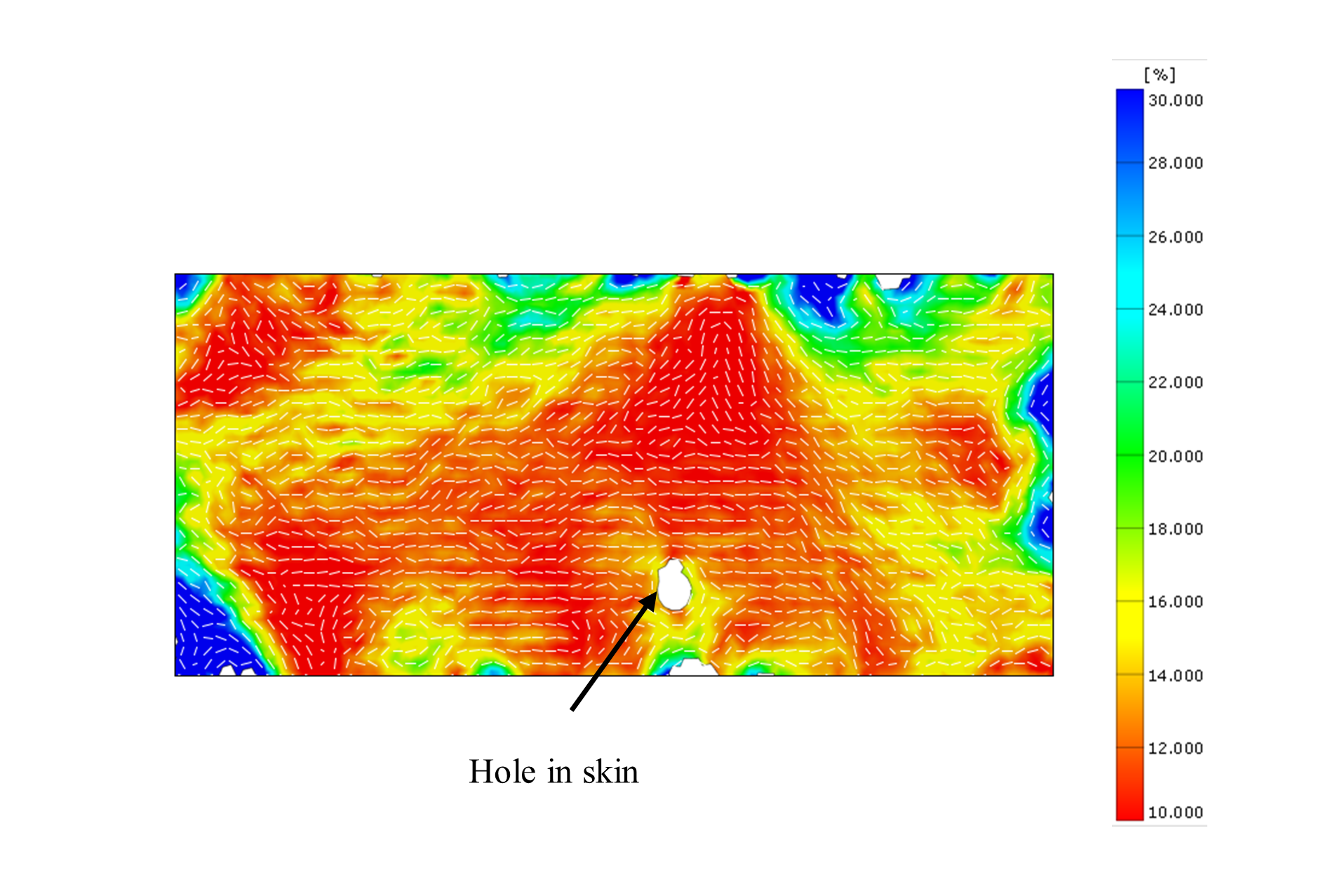

Supplement: S4 Fig — (TIF) [file pone.0343718.s007.tif]

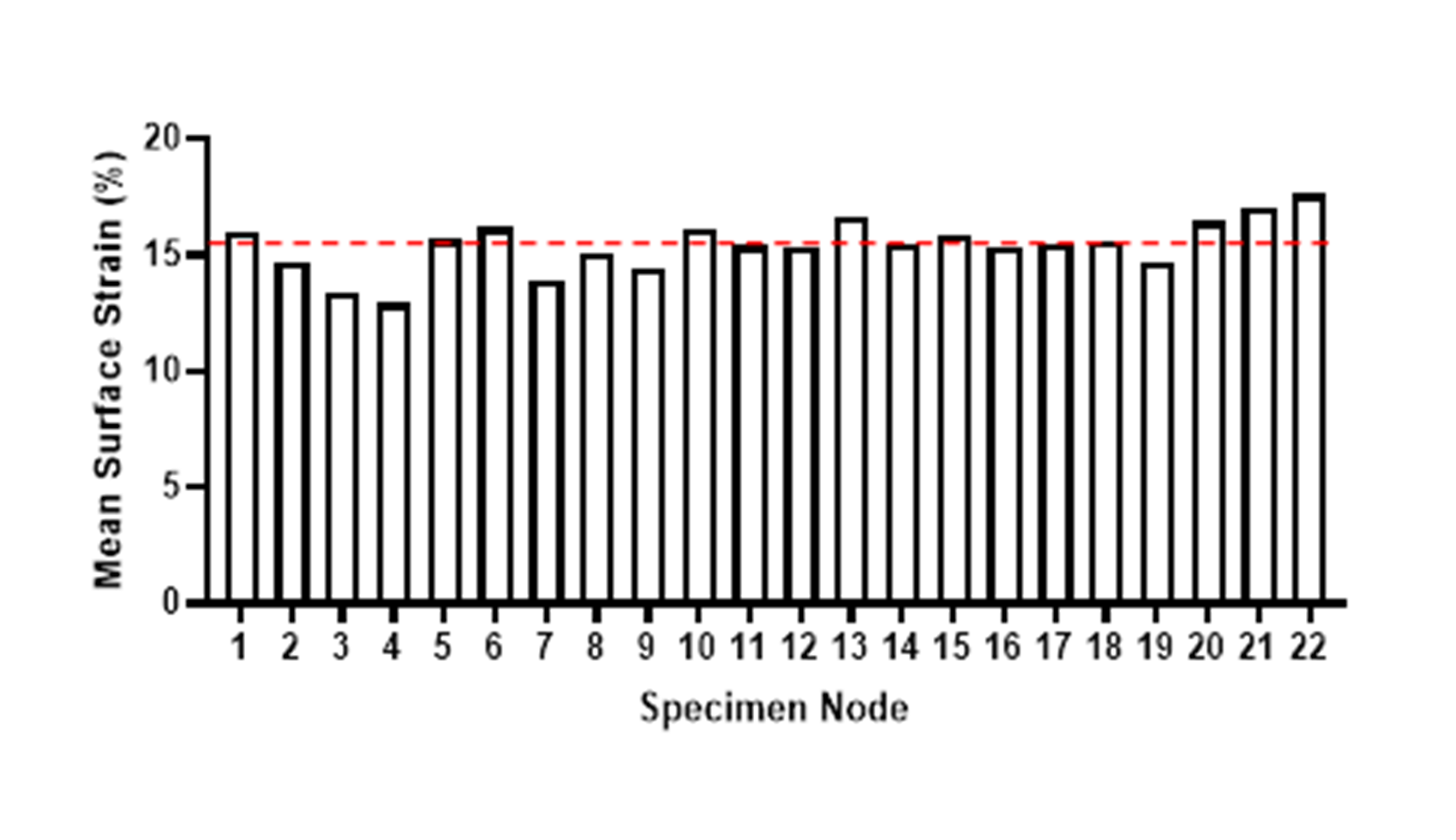

Supplement: S5 Fig — Red line represents the mean (%) major surface strain with all specimen nodes attached. (TIF) [file pone.0343718.s008.tif]
