# Supplementary material for: Multi-axial strain mapping to characterise structure and material properties of the human hip capsule
Source: PLoS One. 2026 Mar 10;21(3):e0343718. doi: 10.1371/journal.pone.0343718 (PMC12974790; doi:10.1371/journal.pone.0343718)
Supplement: S2 Appendix — (PDF) [file pone.0343718.s002.pdf]

S2 Appendix. Example code showing post processing alogirthm for detecting fibrous structures

a. *Determining optimum alignment between strain field matrices and generating a compound matrix (example: optimum alignment in x axis).*

```
%Import first tissue strain field matrix as output
Output = A1=[1 1 1 4; 1 1 1 5; 1 1 1 6];
%Import second tissue strain field matrix as comparator
Comparator = A2 = [2 2 2 3 3 3 4 4 4 5 5 5; 10 10 10 20 20 20 30 30 30 40 40 40;
20 20 20 30 30 30 40 40 40 50 50 50];
% i.e. Comparator is 2x6, while Output is 2x3
% Loop until the last tissue matrix has been added to comparator
% At each stage calculating the mean square intensity
for i=1:(size(Comparator,2)-3)
% New matrices will be named: B1 , B2 , B3 etc.
eval(sprintf('B%d = Output + Comparator(:,(i:(i+size(Output,2)-1)))', i));
eval(sprintf('C%d = sumsqr(B%d)/numel(B%d)', i, i, i));
end
% Repeat operation for comparator and output matrix addition in Y-axis.
% Optimum alignment achieved for alignment position with highest mean squared
intensity
```

b. *Orientation J plugin used for calculating structure tensor matrix and deriving the vector field for calculated compound matrix*

c. *Identifying location and angle of highly orientated structures using the scanning window algorithm*

```
% Define rows and columns of the scanning window
S_rows = 30;
S_cols = 20;
% Define percentage of overlap for horizontal and vertical scan
overlap_horizontal = 0.5;
overlap_vertical = 0.5;
% Define angular step (in degrees) for image rotation
theta_step = 5;
% Import vector field
A = vector field;
% ============================
% Round values of A
A = round(A);
% Preliminary run: find additional rows and columns due to vector field rotation
maxrows=0;
maxcols=0;
for theta = 0 : theta_step : 180-theta_step
   Ar=imrotate(A,-theta);
   if maxrows<size(Ar,1)
      maxrows=size(Ar,1);
   end
```

```
    if maxcols<size(Ar,2)
        maxcols=size(Ar,2);
    end
end
% Adjust additional rows and columns to scan window's dimension
maxrows=((1-overlap_vertical)*S_rows)*ceil((maxrows/((1-
overlap_vertical)*S_rows)));
maxcols=((1-overlap_horizontal)*S_cols)*ceil((maxcols/((1-
overlap_horizontal)*S_cols)));
%--------------
% Scan vector field and store scanned values
for theta = 0 : theta_step : 180-theta_step
    Ar=imrotate(A,-theta);
    I=zeros(maxrows,maxcols);
    I(1:size(Ar,1),1:size(Ar,2))=Ar;
    for i = 1 : (1-overlap_vertical)*S_rows : (maxrows-S_rows+1)
        for j = 1 : (1-overlap_horizontal)*S_cols : (maxcols-S_cols+1)
            eval(['S' num2str(theta) '_' num2str(i) '_' num2str(j) '=I(i:i+(S_rows-
1),j:(j+S_cols-1));']);
        end
    end
end
% Calculate peak values for each scanned window
k=1;
 for theta = 0 : theta_step : 180-theta_step
     for i = 1 : (1-overlap_vertical)*S_rows : (maxrows-S_rows+1)
         for j = 1 : (1-overlap_horizontal)*S_cols : (maxcols-S_cols+1)
            [S_val,jj,kk]=unique(eval(['S' num2str(theta) '_' num2str(i) '_'
num2str(j)]));
            S_freq=accumarray(kk,1);
            B=[S_val S_freq];
            B=sortrows(B,2,'descend');
            %Store angle of rotation, row and column of scan window
            PeakVals(k,1)=theta;
            PeakVals(k,2)=i;
            PeakVals(k,3)=j;
            %Peak value
            PeakVals(k,4)=B(1,1);
            %Peak value's frequency
            PeakVals(k,5)=B(1,2);
            %Peak value's frequency percentage
            PeakVals(k,6)=B(1,2)/numel(kk);
            k=k+1;
         end
     end
 end
```

At the end of the operation, we get a final matrix containing angle of rotation, starting row of scan window, starting column of scan window, peak fibre orientation angle (value), frequency, frequency percentage.
