# Supplementary material for: Multi-axial strain mapping to characterise structure and material properties of the human hip capsule
Source: PLoS One. 2026 Mar 10;21(3):e0343718. doi: 10.1371/journal.pone.0343718 (PMC12974790; doi:10.1371/journal.pone.0343718)
Supplement: S3 Appendix — (PDF) [file pone.0343718.s003.pdf]

S3 Appendix. Sensitivity analyses to verify the performance and robustness of the opto-mechanical characterisation system.

To evaluate the performance of the opto-mechanical characterisation method, four sensitivity studies were performed.

1. *Presence of a biaxial stress distribution verified for an isotropic and anisotropic material*

   The concept of analysing the macro-scale structural organisation and material properties of highly anisotropic materials by imposing homogenous stress distribution was evaluated for isotropic and anisotropic materials. Two elastomeric parts: An isotropic part and an anisotropic part with channels forming the letter "K" with reduced thickness were 3D printed using an Objet500 Connex3 PolyJet 3D printer (Stratasys, Eden Prairie, MN). Both parts were designed to have outer dimensions of 140 mm x 50 mm x 3 mm and printed with a Young's Modulus of 1.5 GPa. The printed parts were speckled using an airbrush technique previously reported by Palanca et al.[56] and mounted onto the device platform using double hook attachments.

2. *The effect of this fishhook gripping method was evaluated by quantifying the Saint-Venant boundary effects in a finite element model.*

   Simulations of the stress distribution field generated by the multi-axial device setup were modelled using finite element software (ABAQUS/Standard 6.13, Dassault Systèmes Simulia Corp., Rhode Island, USA). A model of the 3D printed anisotropic elastomer was implemented with actuator loading conditions and hook attachment points synonymous to the multi-axial testing setup. The material properties were applied as linear elastic properties defined using Young's Modulus and Density parameters used for generating the 3D printed elastomer. The resultant Max Principal strain fields were compared with the

DIC strain field measurements. The effect of the fishhook gripping method was evaluated by quantifying the Saint-Venant boundary effects along the centreline of the specimen. The stress decay along the centreline was quantified using the von Mises stress.

3. *Application of the opto-mechanical characterisation method to another biological tissue*

A human skin sample harvested from the shoulder region of a fresh frozen cadaveric specimen and prepared with a white-on-black speckle technique. A homogenous biaxial stress distribution was applied across the sample with same tissue attachment configuration and loading conditions as detailed previously. The (%) major strain field was calculated, and the macro-scale fibrous structures were analysed.

4. *The performance of the stress distribution field generated by the multi-axial device*

A human skin sample harvested from the shoulder region of a fresh frozen cadaveric specimen and prepared with a white-on-black speckle technique. The mean (%) major strain across the tissue specimen was calculated under a uniform homogenous biaxial stress distribution. A total of 22 tests were run, each time one actuator attachment was left detached from the tissue before running the test. The mean (%) major strain was calculated for each scenario. The mean percentage error is computed and was used to evaluate the characterisation technique.

RESULTS

1. *Presence of a biaxial stress distribution verified for an isotropic and anisotropic material*

The mean (%) major strain across the primary region of the isotropic and anisotropic parts was 3.15% ± 0.4 % and 3.25 % ± 0.6 %, respectively. In comparison, the mean (%) major strain in the channel regions of the anisotropic part was 6.7 % ± 0.2 %, exhibiting

approximately twice the strain for 50% reduction in cross-sectional area in the channel region. The “K” channel region was visually verified from the strain fields (S2 Fig.).

2. *The effect of this fishhook gripping method was evaluated by quantifying the Saint-Venant boundary effects in a finite element model.*

The results indicate that the influence of the local stresses imposed by this gripping technique had diminished at 8 mm away from the grip edge (S3 Fig.). The central region covering an area of 88% between the grips in the circumferential axis and 65% between the grips in the longitudinal axis produced a homogenous stress distribution free of edge effects. Furthermore, the mean (%) Max Principal Strain in the primary region and channel region of the anisotropic part was 3.1 % and 7.2 %, respectively, corroborating with DIC strain field measurements (S2 Fig.).

3. *Application of the opto-mechanical characterisation method to another biological tissue*

The opto-mechanical characterisation method was able to capture skin’s anisotropic nature, displaying the collagen fibre network and widely recognised Langer’s Lines (emphasised by vector field) (S4 Fig.).

4. *The performance of the stress distribution field generated by the multi-axial device*

The leave-one-out analysis showed that the characterisation method was able to predict the major surface strain with a mean squared error of 4% (S5 Fig.).
