# Supplementary material for: Multi-axial strain mapping to characterise structure and material properties of the human hip capsule
Source: PLoS One. 2026 Mar 10;21(3):e0343718. doi: 10.1371/journal.pone.0343718 (PMC12974790; doi:10.1371/journal.pone.0343718)
Supplement: Attachment — Submitted filename: Review of PONE-D-25-14629.pdf [file pone.0343718.s009.pdf]

Review of PONE-D-25-14629

MAJOR COMMENTS

In the abstract, the authors at lines 33-34 say "We experimentally validate our method by applying it to the hip joint capsule", while they say nothing about skin and artificial anisotropic material. Actually, from the main text, it seems that validation is against skin and artificial material, while hip joint capsule is the focus application. Therefore, this passage should be revised and briefly integrated.

The novelty of the proposed technique should be better demonstrated. For example:

i. a detailed comparison is expected vs references (18, 26-28) and (17), overall vs (26-28) and (17) that are cited in Introduction but not in Discussion;
ii. the study is based on the statement "the direct link between the local structural properties and tissue function need to be further investigated (33). This gap…". However, a single, not recent (2015) paper (ref. 33) is not enough to support the whole rationale of this study. Check for a more recent bibliography, here and in general;
iii. definitely, more explicit support should be given in the manuscript to demonstrate the novelty of the technique and its impact. Which are the designed advantages respect to the state of the art? In a dedicated Discussion section, the key-elements – i.e. "biaxial", "homogeneous stress", "anatomy/structure", "mechanical properties", "local", "large field of view" – could be declared, singularly highlighted in their importance and then the authors could demonstrate that their technique, combining those elements, represents an innovation and an advancement respect to the most recent solutions: e.g., refer and compare to:
    - *Microstructure and mechanics of the bovine trachea: Layer specific investigations through SHG imaging and biaxial testing (2022)*
    - *Mechanical, structural, and morphological differences in the iliac arteries (2024)*
    - *Biomechanics of mitral valve leaflets: Second harmonic generation microscopy, biaxial mechanical tests and tissue modelling (2022)*
    - *The regional-dependent biaxial behavior of young and aged mouse skin: A detailed histomechanical characterization, residual strain analysis, and constitutive model (2019)*
    - *Effects of repeated biaxial loads on the creep properties of cardinal ligaments (2017)*

In "2.2 Specimen preparation", the authors speak about nine harvested capsules, but in the rest of the text, it seems that only six are tested. Please, clarify this discrepancy, and comment about the m/f = 6/3 ratio in relation to the homogeneity of the tested group (i.e., hypothesis of no difference between males and females? Or only males tested?).

As for the general aim, the specific aims of paragraph 2.6 "Sensitivity analyses" should be more explicit in the text. That is, for each one of the analyses 1), 2), 3) and 4), to say why and why it is important to do (i.e., what their results implicate).

Related to 2.7 "Statistical analysis", what if data were not normal? It should be taken into consideration; moreover, authors do not say that data resulted normal in 3 "Results"paragraph.

MINOR COMMENTS

Abstract

- Line 37, please briefly specify how strain measures anatomy

Introduction

- An introduction figure could be useful, a sort of graphical abstract
- Line 44, “play” instead of “plays”
- Line 45, check the position of the dot after “(1)” and in the rest of the manuscript
- Line 46, “comprises” instead of “comprise”
- Line 47, “is” instead of “are”
- Lines 54-61 underline the importance of the load-dependent nature of the fibrous collagen network; but this work does not really investigate this concept, i.e. a load-dependent arrangement. Please, clarify
- Line 63, what is “tissue-level”? Please, revise the statement
- Lines 74-76, some comments could be added in relation to contrast-enhanced microtomography (e.g., see https://doi.org/10.3390/biomimetics9080477)
- Line 86, “,” instead of “;”
- Line 90 ,“needs” instead of “need”
- Line 95, please specify what is “this new methodology”
- Line 96, “three” word better than “3” number

Materials and Methods

- Line 115, move “Fig.” before “S2”, renumber figures in order of citation (e.g., S1 should be cited before S2, Fig. 1 before Fig. 2 at line 118 and so on)
- Line 118, “accompanies” instead of “accompany”
- Lines 124-125, 129, 131, “is” instead of “was”, “allows” instead of “allowed”, “consists” instead of “consisted” and “acquires” instead of “acquired”; present tense is better than past tense in describing the system
- Line 138, “nine” better than “9”
- Lines 142-144, please explain why using two alternative longitudinal cuts
- Line 170, “was” instead of “were”
- Lines 170-173, please add the resulting spatial resolution
- Line 204, please specify that “identified” is in the way described in the previous paragraph 2.4
- Line 210, “thicknesses” better than “thickness”
- Line 219, reorder supplementary references (S2 was cited before S1 here). Moreover, S1 file cannot be opened because corrupted, therefore cannot be examined
- Line 223-225, difference between what? Uniaxial and biaxial loading? If effects from shear components are minimal, why using bi-axial loading instead of uniaxial in the proposed set-up? Please, explain better in the text the rationale of this section
- Line 242, remove “and” before “mounted
- Line 248, “.” after specimen

Results

- Line 269, please specify the mean value of applied stress. Moreover, authors speak about homogeneous stress, which seems in contrast with measuring different thicknesses on the capsule (lines 207-210), at fixed force. Please, clarify
- Line 273, “seven” better than “7” (same for line 343)

Discussion

- Lines 352-353, again, the concept of understanding the load-dependent nature (or analogue structure-function relationship) seems mislead in this work. The authors reveal structure by strain, but not strain/load-dependent arrangement. That is, the output seems closer to that of lines 438-439, i.e. characterising the structural anatomy and properties together, than to revealing arrangement of load-dependent fibrous network (line 440). Please, clarify
- Line 368, “were” instead of “was”
- Line 190, check the punctuation position after “(39,41,42,50)” and in the rest of the manuscript (e.g., comma at line 409)
