# Supplementary material for: Multi-axial strain mapping to characterise structure and material properties of the human hip capsule
Source: PLoS One. 2026 Mar 10;21(3):e0343718. doi: 10.1371/journal.pone.0343718 (PMC12974790; doi:10.1371/journal.pone.0343718)
Supplement: Attachment — Submitted filename: Response to reviewers_PLOS.docx [file pone.0343718.s010.docx]

**Response to reviewers’ comments**

We would like to thank the editor and reviewers for their constructive and insightful comments, which have significantly improved the quality and clarity of our manuscript. In response, we have undertaken a comprehensive revision, including substantial rewriting and restructuring of the Introduction, Methods, Sensitivity Analyses, and Discussion. This has strengthened the manuscript considerably. Below, we address all comments in order, with our detailed responses provided in red.

**Reviewer 1**

**Comment #1: Abstract and Introduction in general: The Authors mention the innovative nature of their method but, in the Reviewer’s opinion, this take-home-message can be a bit speculative since, in the end, is a 2D DIC commercial system, with images acquired with a commercial photo camera, equipped on a bi-axial multi-actuator testing machine. The Reviewer suggests rephrasing the abstract/end of the introduction (or where appropriate) giving more evidence to the bi-axial machine instead of describing the setup as an innovative technique.**

Response #1: Thank you. We have revised the Abstract and Introduction to use a more conservative description of our contribution. The revised text clarifies that the experimental setup uses standard equipment, including a commercial biaxial testing device and a 2D DIC system. We now emphasise that the contribution of the study is methodological. The focus is on how a homogeneous biaxial stress field and full field strain mapping are combined to extract collagen architecture across large tissue areas.

We also highlight that the key enabling feature is the controlled biaxial loading rather than the imaging hardware. These revisions ensure that the manuscript reflects the method as an accessible approach built from established tools rather than implying that the instrumentation itself is new.

We have revised the following text in the abstract (line 30) and (line 42):

*[Here we apply a commercial 2D Digital Image Correlation (DIC) system integrated with a biaxial testing machine to quantitatively characterise the fibrous structure of biological materials.]*

*[By combining a biaxial testing machine with commercial 2D DIC system, this study demonstrates a practical and scalable approach for quantifying tissue structure-function relationships across whole tissues.]*

We have revised the following text in the introduction (line 111):

*[Therefore, the aim of this study was to develop and demonstrate a biaxial testing approach that combines multi-axial loading with optical strain measurement to quantify the fibrous anatomy and spatial mechanical properties of complete tissues within a single biaxial test. Rather than introducing new imaging hardware, the contribution of this work lies in leveraging commercially available biaxial actuators and 2D digital image correlation within a unified protocol designed to reveal load-dependent collagen organisation at the centimetre scale. The method was experimentally validated using an artificial anisotropic material and ex vivo skin and was then applied to the human hip joint capsule.]*

**Comment #2: Introduction, lines 62-76: among the different imaging and X-ray/ strain-based techniques please add also the digital volume correlation approach and explain the pros-cons of this technique.**

Response #2: We have added digital volume correlation (DVC) to the list of strain-based imaging techniques in the Introduction. We briefly discuss its advantages, including the ability to measure 3D volumetric strain and internal tissue deformation and its suitability for capturing tissue heterogeneity. We also note its requirements, such as high-resolution CT or X-ray imaging and computational intensity, which can limit throughput.

We have added the following text to the introduction (line 77):

*[DVC offers three-dimensional, volumetric strain and displacement measurements throughout a tissue, enabling assessment of internal deformation under load (26). While DVC provides valuable insights into internal tissue mechanics and heterogeneity, it typically requires high-resolution CT or X-ray imaging and can be computationally intensive.]*

**Comment #3: lines 113-128: The Authors state that the load cells of the arms are 45N which is a quite low end-scale for characterising the mechanical behavior of ligaments up to failure. Please evaluate to add the typical force-strain curves of the hip joint capsule showing where your data falls into that curve.**

Response #3: We agree that a 45 N actuator limit may appear low if a study aims to test ligaments to failure. In our work, however, the tissues are not loaded into the high-stiffness or failure region, and we have now clarified this in the manuscript. Hip capsule force–displacement data reported in Hewitt et al. (2002) show that the transition to high stiffness and failure occurs well above the 40 N loads applied in our protocol. As shown in the newly added comparison figure (S1 Fig.), our loading range remains entirely within the initial linear region of the ligament response. We have added text to the Materials and Methods to explain that the 45 N actuator capacity is appropriate for the anatomical and mechanical characterisation performed in this study.

We have added the following text to the Materials and Methods section (line 128):

*[This load range is appropriate for the present study because the imposed forces remain within the low to mid stiffness region of hip capsule behaviour. Published force–displacement data for the iliofemoral ligament, including those reported by Hewitt et al., show that the transition toward high stiffness and eventual failure occurs well above the 40 N loads applied here. As shown in Supplementary Material (S1 Fig.), where representative curves from Hewitt et al. are plotted alongside the forces generated in this study, our measurements fall within the initial linear region of the capsule response. This confirms that the 45 N actuator capacity is sufficient for accurate anatomical and mechanical characterisation without approaching failure loading.]*

**Comment #4: Please, if you acquire new data, be consistent with the strain-rate used for your tests, otherwise report literature data but explain possible differences with the strain-rate applied between the Authors’ and the literature data.**

**- please clearly report the strain-rate applied for your tests since, being ligaments viscoelastic, this parameter is fundamental to evaluate possible stiffening or softening of the material. Please also state if the strain-rate values used were physiological or not. Please add this data close to the 1 N/s value the Authors reported as speed.**

Response #4: Thank you for highlighting the importance of reporting strain rate in ligament testing. In the revised manuscript, we now explicitly state the approximate strain rate associated with our loading protocol and clarify its relationship to published values. Specifically, we report that the 1 N/s force-controlled loading corresponded to an estimated strain rate of 0.01 s⁻¹, which falls within the low, quasi-static range traditionally used for characterising ligament mechanics.

We also note clearly that this strain rate is non-physiological, consistent with displacement-controlled protocols used in prior capsule studies such as Hewitt et al. and Pieroh et al., which similarly employ slow deformation rates (0.04 mm/s to 0.2 mm/s) to minimise viscoelastic rate-dependent effects. This new text has been added next to the description of the 1 N/s loading rate.

We have added the follow text to the Methods section (line 203):

*[At this loading rate, the resulting tissue deformation corresponded to an approximate strain rate of 0.01 s⁻¹. This value lies within a quasi-static, non-physiological regime and is comparable to the low strain rates used in previous characterisation studies of the hip capsule, including those reported by Hewitt et al. and Pieroh et al. Using a quasi-static strain rate minimises viscoelastic rate effects and enables direct comparison with published capsule mechanics.]*

**Comment #5: Lines 129-136: looking at the image of the system, just one camera was used so the local strain information is just 2D. Please clearly state for the Inexpert Reader and argument how you overcome the problem of possible tissue present in different planes during the acquisition.**

Response #5: We now clarify in the manuscript that a single-camera DIC configuration provides 2D in-plane strain measurements only and does not capture out-of-plane deformation. In our study this was appropriate because the capsule specimens were mounted fully flat on a rigid planar frame and loaded strictly within their plane, substantially minimising any out-of-plane motion. The multi-actuator device applies forces tangentially along the specimen edges, meaning that deformation occurs primarily within the imaging plane, and any through-thickness motion is negligible relative to the camera’s depth sensitivity.

The following text has now been added to the Methods (line 151):

*[A single camera was used for two dimensional DIC, meaning that only in plane strain components were measured. This setup is appropriate for the present study because the hip capsule specimens were mounted fully flat on a rigid frame and loaded strictly within their plane. The applied biaxial forces act tangentially along the specimen boundaries, which minimises out-of-plane deformation and ensures that the measured strain field accurately reflects the in-plane mechanical response of the tissue.]*

**Comment #6: lines 151-155: about the speckle pattern: did the Authors test that the powder and the titanium dioxide did not compromise the natural tissue properties? This information is fundamental to validate our method. Moreover, being a speckle pattern based on particles and powder, how the Authors were sure to not lose locally the correlation during the hydration of the tissue or the possible detachment of the powder. Please clarify also in the manuscript.**

Response #6: The charcoal and titanium dioxide powders used for the DIC pattern are inert and non-reactive and were applied in minimal quantities that do not measurably influence tissue stiffness or surface hydration. To confirm that the pattern did not modify the mechanical response, we compared load–displacement curves obtained using two-point tracking (without a speckle pattern) to those obtained with DIC using the powder-based pattern and observed no detectable differences in the measured behaviour.

To address the concern regarding potential particle detachment or local correlation loss, we added a detailed explanation to the Methods. The capsule specimens were fully hydrated prior to speckle application, after which no additional hydration was applied during testing. The total acquisition time was under 300 s, which further reduces the likelihood of particle detachment. In addition, the flat mounting configuration of the capsule minimises out-of-plane deformation and stabilises the speckle layer, and we did not observe any loss of correlation during testing.

These clarifications have now been incorporated into the manuscript at the corresponding section in the Methods (line 182).

*[To ensure that the charcoal and titanium dioxide particles did not alter the natural mechanical behaviour of the capsule, we verified that these materials are inert, non-reactive and applied in quantities that do not measurably stiffen, hydrate or otherwise modify the capsule surface. The tissue was fully hydrated prior to speckle application, and no further hydration was applied during testing. The total acquisition time was under 300 s, which limited any risk of particle detachment or changes to the tissue surface. We also confirmed that the speckle pattern did not influence the mechanical response by comparing load–displacement curves obtained with two-point tracking, without a speckle pattern, against curves acquired with DIC using the powder-based pattern and found no detectable differences. The stability of the speckle pattern was further supported by the flat mounting configuration of the capsule, which minimised out-of-plane motion and prevented local loss of particle adhesion or correlation during deformation.]*

**Comment #7: lines 170-175: To have an overall idea of the systematic and random error of the DIC investigation did the Authors performed a zero-strain analysis? Please clarify in the manuscript.**

Response #7: Yes, a zero-strain analysis was performed to assess both systematic and random error in the DIC measurements. A stationary capsule specimen was imaged under identical optical and correlation settings, and the resulting strain field was quantified. The analysis showed negligible mean strain and low random noise within the expected range for facet and overlap sizes used. We have now added a description of this procedure and the corresponding error bounds to the Methods section (line 214).

*[To quantify the systematic and random error of the DIC system, a zero-strain analysis was performed by acquiring images of a stationary capsule specimen under identical optical, lighting, and correlation settings. The resulting strain field showed mean values close to zero (approximately 0.02%) with random noise on the order of 0.10 %, consistent with expected precision for the selected facet and overlap sizes. This confirmed that the DIC configuration did not introduce measurable artificial strain during acquisition.]*

**Comment #8: general remark: is important to clearly mention in the manuscript, for the Inexpert Reader, that all the local strains described in the text via this DIC system are only 2D. To obtain a 3D DIC investigation a minimum of 2 cameras are needed.**

Response #8: We agree and have now added an explicit statement in the manuscript that all strain measurements are 2D because they are derived from a single-camera DIC system. We also note that 3D strain measurement requires at least two synchronised cameras.

This clarification has been added to both the Methods section (line 221) and the figure description of the experimental setup.

*[All strain fields reported in this study represent in-plane deformation measured using a single-camera 2D DIC system. Full three-dimensional strain mapping would require a stereoscopic configuration with at least two cameras, which was not used in the present work.]*

**Reviewer 2**

**Comment #1: In the abstract, the authors at lines 33–34 say “We experimentally validate our method by applying it to the hip joint capsule”, while they say nothing about skin and artificial anisotropic material. Actually, from the main text, it seems that validation is against skin and artificial material, while hip joint capsule is the focus application. Therefore, this passage should be revised and briefly integrated.**

Response #1: The abstract has been revised to clarify that the method was first validated using an artificial anisotropic material and a skin sample before being applied to the human hip joint capsule as the main application. This ensures consistency with the experimental description in the main text.

We have edited the abstract to make this clear (line 35):

*[The method was validated using an artificial anisotropic material and ex vivo skin, and was then applied to the human hip joint capsule to demonstrate its ability to characterise complex collagen networks.]*

**Comment #2: A detailed comparison is expected vs references (18, 26–28) and (17), overall vs (26–28) and (17) that are cited in the Introduction but not in the Discussion.**

Response #2: We thank the reviewer for this helpful comment. The discussion now includes a detailed comparison with the optical–mechanical systems cited in the Introduction (references 17, 18, 26–28), which employ SALS, pSFDI, and PLI techniques combined with biaxial testing to study collagen fibre organisation under load. This addition clarifies how the proposed DIC-based framework differs in scale, implementation, and accessibility from these systems.

The revised paragraph now reads as follows (line 478–495):

*[Optical–mechanical systems have significantly advanced understanding of collagen architecture under load by coupling imaging modalities with biaxial or uniaxial testing. Early work by Sacks and colleagues (26, 28) combined biaxial mechanical testing with small-angle light scattering (SALS) to quantify collagen fibre orientation and realignment in soft tissues. Subsequent systems, including those described by Yang et al. (20), Blair et al. (61), Jett et al. (18), and Laurence et al. (27), expanded this principle using advanced imaging techniques such as polarised spatial frequency domain imaging (pSFDI) and polarised light imaging (PLI) to characterise collagen architecture during deformation. More recent studies have employed second-harmonic generation (SHG) microscopy to link microstructure and function, including layer-specific investigations in the bovine trachea (37), mitral valve leaflets (38), and regional mechanical–structural characterisation of mouse skin (39). Related biaxial investigations have also examined the creep behaviour of cardinal ligaments (40) and structural differences in the iliac arteries (41). These techniques achieve high angular precision but rely on laser optics, polarisation control, and thin, transparent samples, which restrict their field of view to sub-millimetre or millimetre-scale regions. In contrast, the present DIC-based framework infers structural orientation from surface strain fields across centimetre-scale areas under a homogeneous biaxial stress, eliminating the need for specialised optical hardware. This enables quantitative assessment of fibre orientation and regional stiffness while revealing the functional anatomy of the tissue through its load dependent fibrous network. The method therefore complements SALS, pSFDI, and PLI systems by extending their insights to the tissue scale within a single, accessible experiment.]*

**Comment #3: The study is based on the statement “the direct link between the local structural properties and tissue function need to be further investigated (33). This gap…”. However, a single, not recent (2015) paper (ref. 33) is not enough to support the whole rationale of this study. Check for a more recent bibliography, here and in general.**

Response #3: Thank you - we agree. We have expanded the introduction to cite recent work that links local collagen architecture to mechanical behaviour under load across multiple tissues. We replaced the single citation to ref. 33 with a set of recent sources and clarified why a tissue scale, load dependent mapping method is still needed.

The outdated single reference (33) has been replaced with a broader and more current bibliography: refs (30,35–41).

The revised text now reads (line 94-101):

*[Recent studies coupling biaxial mechanical testing with microstructural imaging have revealed strong regional dependencies between collagen orientation, dispersion, and mechanical response in tissues such as arteries, valve leaflets, and skin (34–41). However, such systems often require specialised microscopy setups and are limited to small fields of view. This gap underscores the need for a method that combines multi-axial loading, homogeneous stress fields, and digital image correlation (DIC) to provide centimetre-scale, spatially resolved strain mapping that directly links local structure to mechanical function. Such an approach can also be used alongside microstructural imaging methods to bridge tissue-scale strain behaviour with underlying fibre architecture.]*

**Comment #4: definitely, more explicit support should be given in the manuscript to demonstrate the novelty of the technique and its impact. Which are the designed advantages respect to the state of the art? In a dedicated Discussion section, the key-elements – i.e. “biaxial”, “homogeneous stress”, “anatomy/structure”, “mechanical properties”, “local”, “large field of view” – could be declared, singularly highlighted in their importance and then the authors could demonstrate that their technique, combining those elements, represents an innovation and an advancement respect to the most recent solutions: e.g., refer and compare to:**

- **Microstructure and mechanics of the bovine trachea: Layer specific investigations through SHG imaging and biaxial testing (2022)**
- **Mechanical, structural, and morphological differences in the iliac arteries (2024)**
- **Biomechanics of mitral valve leaflets: Second harmonic generation microscopy, biaxial mechanical tests and tissue modelling (2022)**
- **The regional-dependent biaxial behavior of young and aged mouse skin: A detailed histomechanical characterization, residual strain analysis, and constitutive model (2019)**
- **Effects of repeated biaxial loads on the creep properties of cardinal ligaments (2017)**

Response #4: Thank you for highlighting this. The discussion has been revised to explicitly describe the novelty of the proposed technique and how it complements existing optical–mechanical approaches. A new paragraph explains the key methodological elements of the work, including biaxial loading, homogeneous stress verification, digital image correlation, local spatial resolution, functional mapping of anatomical structures, and a large field of view. The text clarifies how combining these elements in a single experimental framework provides a unified and accessible method for linking local structure and mechanics across complete tissues.

The following text has been added to the discussion (line 461-477):

*[The novelty of this study lies in the integration of several key methodological elements within a single, accessible framework. The approach combines biaxial mechanical loading, homogeneous stress verification, digital image correlation (DIC), and centimetre-scale imaging to enable simultaneous characterisation of structure and function without disrupting native tissue architecture. Biaxial loading provides a controlled mechanical environment that captures multi-directional fibre orientations and their spatial distribution across the sample. Establishing a homogeneous stress field ensures that regional strain differences reflect intrinsic material behaviour rather than boundary artefacts. The use of DIC provides local spatial resolution, enabling detailed mapping of strain fields and quantitative inference of collagen fibre orientation and recruitment. By preserving the anatomical continuity of the specimen and avoiding sectioning or fixation, the method maintains structural relevance and links regional fibre organisation directly to mechanical function. The large field of view bridges the gap between microscopic imaging and bulk mechanical testing, while the simultaneous derivation of local tangent moduli provides direct quantification of mechanical properties within the same experiment. Each of these elements has been applied independently in earlier studies, but their integration here creates a unified experimental system capable of mapping the local structure–function relationship across entire tissues. This combination represents a significant advance over conventional testing approaches.]*

The discussion was also expanded to include a comparison with recent optical–mechanical studies, showing how the present method complements these systems. The following text was added (line 478-495):

*[Optical–mechanical systems have significantly advanced understanding of collagen architecture under load by coupling imaging modalities with biaxial or uniaxial testing. Early work by Sacks and colleagues (26, 28) combined biaxial mechanical testing with small-angle light scattering (SALS) to quantify collagen fibre orientation and realignment in soft tissues. Subsequent systems, including those described by Yang et al. (20), Blair et al. (61), Jett et al. (18), and Laurence et al. (27), expanded this principle using advanced imaging techniques such as polarised spatial frequency domain imaging (pSFDI) and polarised light imaging (PLI) to characterise collagen architecture during deformation. More recent studies have employed second-harmonic generation (SHG) microscopy to link microstructure and function, including layer-specific investigations in the bovine trachea (37), mitral valve leaflets (38), and regional mechanical–structural characterisation of mouse skin (39). Related biaxial investigations have also examined the creep behaviour of cardinal ligaments (40) and structural differences in the iliac arteries (41). These techniques achieve high angular precision but rely on laser optics, polarisation control, and thin, transparent samples, which restrict their field of view to sub-millimetre or millimetre-scale regions. In contrast, the present DIC-based framework infers structural orientation from surface strain fields across centimetre-scale areas under a homogeneous biaxial stress, eliminating the need for specialised optical hardware. This enables quantitative assessment of fibre orientation and regional stiffness while revealing the functional anatomy of the tissue through its load dependent fibrous network. The method therefore complements SALS, pSFDI, and PLI systems by extending their insights to the tissue scale within a single, accessible experiment.]*

**Comment #5: In “2.2 Specimen preparation”, the authors speak about nine harvested capsules, but in the rest of the text, it seems that only six are tested. Please, clarify this discrepancy, and comment about the m/f = 6/3 ratio in relation to the homogeneity of the tested group (i.e., hypothesis of no difference between males and females? Or only males tested?).**

Thank you for pointing out this inconsistency. A total of nine hip capsules were harvested and used in both the anatomical characterisation and mechanical testing. The reference to six capsules was a typographical error, and all relevant sections and figure captions have been corrected to n = 9.

Regarding the sex distribution (m/f = 6/3), previous work has shown no significant sex-related differences in tensile properties of capsular ligaments within this age range. Based on this evidence, male and female specimens were pooled for analysis, now stated clearly in Section 2.2 (line 163):

*[Both male and female specimens were pooled for analysis, as previous work has reported no significant sex-related differences in the tensile properties of capsular ligaments within this age range.]*

**Comment #6: As for the general aim, the specific aims of paragraph 2.6 “Sensitivity analyses” should be more explicit in the text. That is, for each one of the analyses 1), 2), 3) and 4), to say why and why it is important to do (i.e., what their results implicate).**

Response #6: Thank you for this suggestion. Section 2.6 (*Sensitivity analyses*) has been revised to include explicit aims and rationale for each of the four analyses, clarifying their importance in validating the robustness and reliability of the testing setup.

The following text was added to Section 2.6 (line 293-332):

*[Four sensitivity analyses were conducted to verify the performance and robustness of the opto-mechanical characterisation system.]*

*[(1) Verification of homogeneous biaxial stress generation: This analysis confirmed that the actuator array can generate a uniform biaxial stress field across the specimen surface, ensuring that strain measurements represent material response rather than non-uniform loading.]*

*[(2) Assessment of boundary and gripping effects: This analysis determined whether the gripping approach or edge constraints altered stress homogeneity, confirming that local strain variations within biological specimens were intrinsic rather than artefacts of boundary conditions.]*

*[(3) Demonstration of applicability to other biological tissues: This analysis demonstrated the versatility of the technique for different planar collagenous tissues such as skin, fascia, and membranes.]*

*[(4) Evaluation of robustness through actuator removal testing: This analysis showed that the stress distribution field and strain measurements remain stable even with minor deviations in actuator loading, confirming the system’s robustness.]*

*These analyses verified that the testing system produces homogeneous loading and is not affected by boundary conditions along the specimen centreline. They also confirmed that the method can be applied to different soft tissues and maintains stable performance even when actuation symmetry is altered.*

**Comment #7: Related to 2.7 “Statistical analysis”, what if data were not normal? It should be taken into consideration; moreover, authors do not say that data resulted normal in 3 “Results”paragraph.**

Response #7: The *Statistical analysis* section has been revised to clarify how non-normal data would be handled, and the *Results* section now explicitly states that data were confirmed to be normally distributed before parametric testing.

The following sentence was added at the end of section 2.7 Statistical analysis (line 337):

*[If normality was not satisfied, equivalent non-parametric tests (Friedman test with Dunn’s multiple-comparison post hoc) were applied.]*

In addition, the Results section under section 3.2 “Tangent modulus and cross-sectional area of capsular ligaments” now includes the following sentence (line 382):

*[All datasets passed normality testing (Shapiro–Wilk, p > 0.05), confirming that the use of parametric analyses was appropriate.]*

**Minor Comments (Introduction)**

**Comment #8: An introduction figure could be useful, a sort of graphical abstract**

Response #8: Thank you for the suggestion. To improve clarity and provide readers with an overview of the workflow, we have updated Figure 3 to include more in-depth detail of the opto-mechanical method. We have added explanatory details for each stage (stress application, strain extraction, strain field alignment, tensor computation, structure identification).

**Comment #9: In the abstract, please briefly specify how strain measures anatomy.**

Response #9: We added a sentence explaining that, under a homogeneous biaxial load, collagen bundles deform less than the surrounding tissue. As a result, low-strain bands and their directions reveal the locations and orientations of the ligaments.

Revised abstract sentence added (line 33):

*[Under this loading condition, collagen bundles deform less than surrounding tissue, so low-strain bands and their principal directions indicate ligament locations and orientations.]*

**Comment #10: Line 44, “play” instead of “plays”**

Response #10: We have corrected the wording as suggested. “plays” has been changed to “play” in the Introduction (line 46).

**Comment #11: Line 45, check the position of the dot after “(1)” and in the rest of the manuscript**

Response #11: Thank you for pointing this out. We have reviewed the manuscript and corrected the placement of full stops throughout the text.

**Comment #12: Line 46, “comprises” instead of “comprise”**

Response #12: Thank you. We have corrected from “comprise” to “comprises” in the revised manuscript (line 48).

**Comment #13: Line 47, “is” instead of “are”**

Response #13: We have corrected “are” to “is” in the revised manuscript.

**Comment #14: Lines 54-61 underline the importance of the load-dependent nature of the fibrous collagen network; but this work does not really investigate this concept, i.e. a load-dependent arrangement. Please, clarify**

Response #14: Thank you for highlighting this. We agree. We revised the paragraph to clarify that the study does not assess changes in collagen architecture at different load levels. Instead, the method quantifies the fibrous network under a single controlled biaxial load, providing a functionally relevant structural characterisation. We have edited this paragraph (line 53-61):

*[Previous studies have typically examined biological tissues in an unloaded state or by chemical fixation in a loaded state, often limiting the ability to relate observed collagen architecture to the mechanical role of the fibres during loading (11,12). Fibre recruitment occurs only when the tissue is placed under tension, so assessing collagen architecture while the tissue is loaded provides a more functionally relevant description of the fibrous network (13). This perspective is important for interpreting how alterations in collagen structure contribute to diseases such as ligament injury (14), liver fibrosis (15), cerebral aneurysms (16), and stenosis in heart valve leaflets (2). In this study, our aim is not to examine how the collagen network changes with different load levels, but rather to quantify its organisation when subjected to a controlled biaxial stress field.]*

**Comment #15: Line 63, what is “tissue-level”? Please, revise the statement**

Response #15: Thank you. We have revised the sentence to clarify the meaning and avoid the ambiguous term “tissue-level.”

The updated text now reads (line 63):

*[“Many imaging and characterisation techniques have been used to study the macroscopic and microscopic organisation of soft collagenous tissues, such as small-angle light scattering (SALS) (17)…]*

**Comment #16: Lines 74-76, some comments could be added in relation to contrast-enhanced microtomography (e.g., see** [**https://doi.org/10.3390/biomimetics9080477**](https://doi.org/10.3390/biomimetics9080477)**)**

Comment #16: Thank you. We have now included contrast enhanced microtomography in our introduction to demonstrate the wider range of techniques. The following has been added to the text (line 71):

*[Contrast-enhanced µCT improves soft-tissue visibility through staining, although achieving reliable contrast can be challenging and preparation times are often long.]*

**Comment #17: Line 86, “,” instead of “;”**

Response #17: Thank you. We have changed “;” to “,”.

**Comment #18: Line 90 ,“needs” instead of “need”**

Response #18: Thank you. We have changed “need” to “needs”.

**Comment #19: Line 95, please specify what is “this new methodology”**

Response #19: Thank you. We clarified the sentence by explicitly naming the method. The text now refers to “the proposed opto-mechanical characterisation methodology”.

The text in the introduction (line 102) has been changed:

*[The characterisation of tissues with complex collagen structures, such as the hip capsule, provides a good platform for developing and testing the proposed opto-mechanical characterisation methodology.]*

**Comment #20: Line 96, “three” word better than “3” number**

Response #20: Thank you. We have changed “3” to “three”.

**Minor Comments (Materials and Methods)**

**Comment #21: Line 115, move “Fig.” before “S2”, renumber figures in order of citation (e.g., S1 should be cited before S2, Fig. 1 before Fig. 2 at line 118 and so on)**

Response #21: The citation formatting has been corrected so that “Fig.” now precedes “S2,” and all figures (main and supplementary) have been renumbered to follow the order of first citation throughout the manuscript. All corresponding in-text references have been updated accordingly.

**Comment #22: Line 118, “accompanies” instead of “accompany”**

Response #22: Thank you. We have corrected “accompany” to “accompanies” in the manuscript (line 123).

**Comment #23: Lines 124-125, 129, 131, “is” instead of “was”, “allows” instead of “allowed”, “consists” instead of “consisted” and “acquires” instead of “acquired”; present tense is better than past tense in describing the system**

Response #23: Thank you. The methods section has been revised to consistently use the present tense when describing the experimental system. Specifically, “was” has been changed to “is”, “allowed” to “allows”, “consisted” to “consists”, and “acquired” to “acquires”.

**Comment #24: Line 138, “nine” better than “9”**

Response #24: Thank you. We have changed “9” to “nine”.

**Comment #25: Lines 142-144, please explain why using two alternative longitudinal cuts**

Response #25: We have now clarified why two alternative longitudinal cut locations were used. Specifically, we added the following sentence to the Specimen preparation section (line 172):

*[Two longitudinal cut locations were used so that anatomical information missing near one cut region, where strain data cannot be captured, could be recovered from the alternate region.]*

**Comment #26: Line 170, “was” instead of “were”**

Response #26: Thank you - we have made this change.

**Comment #27: Lines 170-173, please add the resulting spatial resolution**

Response #27: Thank you. We have now added the resulting spatial strain resolution to the Methods section.

A sentence has been added Section 2.3 (line 214):

*[This resulted in an effective in-plane spatial strain resolution of approximately 0.45 mm.]*

**Comment #28: Line 204, please specify that “identified” is in the way described in the previous paragraph 2.4**

Response #28: We have now clarified that the term “identified” refers specifically to the strain-based detection method described in Section 2.4.

The sentence has been revised (line 253):

*[The mechanical properties of the structures identified using the strain-based method described in Section 2.4 were extracted in a single test by application of a homogeneous biaxial stress distribution.]*

**Comment #29: Line 210, “thicknesses” better than “thickness”**

Response #29: Thank you. We have made this change (page line 219).

**Comment #30: Line 219, reorder supplementary references (S2 was cited before S1 here). Moreover, S1 file cannot be opened because corrupted, therefore cannot be examined**

Response #30: The supplementary reference order has now been corrected so that S1 is cited before S2. We identified an issue with the S1 file and have reuploaded a new version.

**Comment #31: Line 223-225, difference between what? Uniaxial and biaxial loading? If effects from shear components are minimal, why using bi-axial loading instead of uniaxial in the proposed set-up? Please, explain better in the text the rationale of this section**

Response #31: Thank you for highlighting this. We have clarified in the manuscript that this comparison was made between uniaxial and biaxial loading, and that the purpose was to confirm that shear did not substantially influence strain measurements along each ligament.

We also added a brief explanation that despite minimal shear effects, biaxial loading is essential for generating a homogeneous stress field that engages all fibrous structures simultaneously, which is required for ligament identification and single-test property extraction. We have included the below text (line 275):

*[The difference in (%) strain between the uniaxial and biaxial loading scenarios was less than 5%, indicating that shear components did not substantially influence the strain measured along each ligament. This comparison was performed only to confirm that shear effects did not confound strain extraction along the ligament axis. Biaxial loading remains essential for the proposed method because the capsule contains multiple fibre families with different orientations, and only a homogeneous biaxial stress field can uniformly load the entire fibrous network and reveal its spatial anatomy.]*

**Comment #32: Line 242, remove “and” before “mounted**

Response #33: Thank you. We have removed “and” from the sentence.

**Comment #33: Line 248, “.” after specimen**

Response #33: Thank you. We have made this change.

**Minor comments (Results)**

**Comment #34: Line 269, please specify the mean value of applied stress. Moreover, authors speak about homogeneous stress, which seems in contrast with measuring different thicknesses on the capsule (lines 207-210), at fixed force. Please, clarify**

Response #34: We have now specified the mean applied stress in the Results section as approximately 1.2 MPa, calculated from the peak load and the mean ligament cross-sectional area derived from Table 1. We have added a sentence to Section 3.1 (line 344):

*[Under the imposed loading condition, the mean applied stress across the capsule surface was approximately 1.2 MPa.]*

We have also added text explaining that thickness and width measurements are not used for anatomical mapping but required later when computing ligament-specific stress and tangent modulus, where true cross-sectional area is necessary. The following sentence has been added to Section 2.5 (line 260):

*[These dimensions were not used during anatomical characterisation but were required later to compute ligament-specific stress and tangent modulus, where true cross-sectional area is needed.]*

**Comment #35: Line 273, “seven” better than “7” (same for line 343)**

Response #35: Thank you. We have made this change.

**Minor comments (Discussion)**

**Comment #36: Lines 352-353, again, the concept of understanding the load-dependent nature (or analogue structure-function relationship) seems mislead in this work. The authors reveal structure by strain, but not strain/load-dependent arrangement. That is, the output seems closer to that of lines 438-439, i.e. characterising the structural anatomy and properties together, than to revealing arrangement of load-dependent fibrous network (line 440). Please, clarify**

Response #36: Thank you for pointing this out. We agree this is misleading. Our method identifies fibrous anatomy using strain as a structural marker under a single, controlled biaxial stress field, not as a function of varying load. To address this, we have revised the relevant sentences throughout the manuscript to avoid implying load-dependent reorganisation:

Line 461: *[The novelty of this study lies in the integration of several key methodological elements within a single, accessible framework. The approach combines biaxial mechanical loading, homogeneous stress verification, digital image correlation (DIC), and centimetre-scale imaging to enable simultaneous characterisation of structure and function without disrupting native tissue architecture.]*

Line 517: *[Although high-resolution optical techniques such as SHG and confocal microscopy, and PLI (18,66) remain valuable for microstructural insight, their small imaging windows limit whole-tissue assessment. The current method provides centimetre-scale, single-test characterisation that can be integrated with microstructural imaging in a complementary multiscale framework.]*

Line 536: *[Application to the hip capsule revealed the complexity and regional organisation of its fibrous network, providing new insights into regional mechanical roles.]*

**Comment #37: Line 368, “were” instead of “was”**

Response #37: Thank you. We have made this change.

**Comment #38: Line 190, check the punctuation position after “(39,41,42,50)” and in the rest of the manuscript (e.g., comma at line 409)**

Response #38: Thank you. We have made these changes and have checked the punctuation throughout the manuscript.
